# Analyzing human decisions in IGRT of head-and-neck cancer patients to teach image registration algorithms what experts know

**DOI:** 10.1186/s13014-017-0842-8

**Published:** 2017-06-21

**Authors:** Eva Maria Stoiber, Nina Bougatf, Hendrik Teske, Christian Bierstedt, Dieter Oetzel, Jürgen Debus, Rolf Bendl, Kristina Giske

**Affiliations:** 1 Division of Medical Physics in Radiation Oncology, German Cancer Research Center (DKFZ), INF 280, 69120 Heidelberg, Germany; 2 National Center for Radiation Research in Oncology (NCRO), Heidelberg Institute for Radiation Oncology (HIRO), Heidelberg, Germany; 3 Department of Radiation Oncology, Heidelberg University Hospital, Heidelberg, Germany; 4 Faculty of Computer Science, Heilbronn University, Heilbronn, Germany

**Keywords:** Head-and-neck cancer, Deforming anatomy, Rigid image registration, Knowledge-based IGRT, Positioning correction

## Abstract

**Background:**

In IGRT of deformable head-and-neck anatomy, patient setup corrections are derived by rigid registration methods. In practice, experienced radiation therapists often correct the resulting vectors, thus indicating a different prioritization of alignment of local structures. Purpose of this study is to transfer the knowledge experts apply when correcting the automatically generated result (pre-match) to automated registration.

**Methods:**

Datasets of 25 head-and-neck-cancer patients with daily CBCTs and corresponding approved setup correction vectors were analyzed. Local similarity measures were evaluated to identify the criteria for human corrections with regard to alignment quality, analogous to the radiomics approach. Clustering of similarity improvement patterns is applied to reveal priorities in the alignment quality.

**Results:**

The radiation therapists prioritized to align the spinal cord closest to the high-dose area. Both target volumes followed with second and third highest priority. The bony pre-match influenced the human correction along the crania-caudal axis. Based on the extracted priorities, a new rigid registration procedure is constructed which is capable of reproducing the corrections of experts.

**Conclusions:**

The proposed approach extracts knowledge of experts performing IGRT corrections to enable new rigid registration methods that are capable of mimicking human decisions. In the future, the deduction of knowledge-based corrections for different cohorts can be established automating such supervised learning approaches.

## Background

Image-guided radiotherapy (IGRT) is widely used for treatment of head-and-neck cancer (HNC) patients [1, 2]. Put simply, IGRT is a process with two main components: An automatic component, which involves the acquisition and registration of images, and a manual one, that involves experts to review the images and approve the correction vector [3]. In daily practice, radiation therapists who operate the linear accelerator usually perform this expert work.

In the registration process, the acquired cone-beam CT (CBCT) is rigidly registered to the planning-CT to calculate the couch correction vector. The registration result is also called the “pre-match”. The resulting vector and rotations can be used to correct for patient positioning variations by adjusting the treatment couch prior to treatment. Yet, in the presence of anatomical deformations, an optimal alignment of all deformed structures is not possible [4] and therefore the deduction of the best rigid couch correction is ambiguous [5]. Different strategies to cope with this issue were proposed [6, 7], but so far no standard approach exists to cope with this issue in daily practice. Additionally, commercially available tools result in significantly differing correction vectors for the same input data [8]. Thus, carefully reviewing the pre-match is essential in case of the highly flexible head-and-neck site and correcting it in consideration of the anatomical region that should be aligned best.

Aim of this proof-of-principle study is to present an approach on how to extract the prioritization criteria which experienced radiation therapists apply when correcting the pre-match. These criteria are extracted from the finally accepted alignment. This study additionally proposes a knowledge-based registration approach that automatically generates a pre-match, with the goal of not needing further time-consuming expert correction.

## Methods

### Patient data

The CBCT scans of 25 HNC patients were analyzed retrospectively. Thirteen patients were treated for hypopharyngeal cancer and twelve patients for oropharyngeal cancer. Each patient received 25–40 kV-CBCT scans (757 in total). Planning-CTs and CBCTs had a resolution of (0.97x0.97x3) mm and (1x1x1) mm, respectively. All patients were treated with IMRT or VMAT (Elekta Synergy, Crawley, UK). A simultaneous integrated boost technique was used with two CTVs delineated. The therapeutic CTV (tCTV) encompassed the (pre-surgical) gross tumor volume. The prophylactic CTV (pCTV) enclosed the upper and lower cervical and supraclavicular lymph nodes. Both CTVs were extended by a CTV-to-PTV-margin of 3 mm to define the corresponding PTVs (tPTV and pPTV). All anonymized patient data – image scans, RT structure sets, and positioning information – were retrieved from the HIRO Research Database [9]. Informed consent was obtained from all patients for use of anonymized data in retrospective studies.

### Onsite IGRT workflow

Prior to the first treatment fraction the radiation therapist selects a clipping box on the planning-CT for rigid registration. This box usually remains unchanged during the treatment course and according to the protocol that guides the radiation therapists (RT) includes the volume of interest (VOI) “pPTV” for an optimal bony match computed by the Elekta XVI software package. The match results in a correction vector with six degrees of freedom. Yet, in the absence of a hexapod table, the rotational components are omitted and the fusion of the images is updated. The pre-match is then reviewed by the radiation therapist in all three planes and manually corrected if considered necessary. As only bony pre-match was established in the present protocol, typically nearly all fractions require expert corrections.

### Classification of alignment quality

The quality of the image alignment between the planning-CT and the CBCTs is quantified in terms of intensity-based similarity measures. Because experts are expected to pay attention to the alignment of single VOIs, e.g. the spinal cord, VOI-focused similarity is assessed within certain VOI contours. Global and local similarity measures were calculated. Local measures were calculated in all delineated VOIs, including those present in all patients: pPTV, tPTV, spinal cord, right and left parotid gland, skin, brainstem, and upper part of the lungs. To include the image gradient at the edges of the delineated organs, all VOIs were isotropically expanded by a margin of 3 mm for sampling of all similarity measures. Mutual information (MI) was selected as similarity measure for this analysis.

### Competing methods to extract IGRT corrections

Expert-generated alignments T_RT_ are read out from the central research database [9]. Three competing rigid registration processes for all image pairs generate three further alignments: All three registration approaches are based on an in-house developed registration pipeline implemented in our in-house treatment planning system [10]. MI was chosen as similarity measure in the simplex downhill optimization with only translational degrees of freedom for all registration runs. The first registration aligns bones (T_BONY_) without a registration box. This is achieved by suppressing all HU values lower than the threshold for bones in the planning CT in the joint histogram, which is the basis for the MI calculation. The second and third registrations use rectangular registration boxes and also include soft tissue. The registration box is placed around the large VOI “pPTV” or the small VOI “tPTV”, resulting in two different proposals for correction vectors (T_pPTV_ and T_tPTV_). All transformations were calculated in respect to the initial alignment (T_LASER_).

### Data analysis and construction of a knowledge-based registration approach

Comparison of all competing strategies to generate correction vectors is performed on the residual shift deviations in respect to the vectors accepted by the human experts. The distribution of these differences, represented by their mean

$$ {\varDelta^{Fi}}_{x, y, z}=<\kern0.5em \parallel {T}_{T YPE}^{Fi}-{T}_{RT}^{Fi}\parallel \kern0.5em {>}_{x, y, z} $$

and standard deviation (SD), indicates the distance metric to the correction favored by experts. Fi indicates the i-th fraction and x,y,z the spatial components of the 3-dimensional correction vector for any type TYPE of the correction strategy (e.g. BONY or pPTV). Along to the distribution also the Pearson’s correlation ratio is calculated to support the similarity between the distributions of the vector components.

In order to analyze the expert approved corrections and identify criteria for human decisions, a clustering analysis for structure preference patterns over all fractions was performed. Hereby, we omitted the intensity of improvement or degradation and defined the patterns for cluster labels by

$$ {\left\{{S}_{VOIj}\right\}}^{Fi}=\left\{\begin{array}{c}\hfill 1,\Delta M{I}_{VOIj}^{Fi}\ge 0\hfill \\ {}\hfill 0, else\hfill \end{array}\right. $$.

The Gower General Similarity Coefficient [11] was used as distance measure for clustering.

### Knowledge-based rigid registration scheme

To automatically reproduce the experts’ corrections, a standard rigid registration approach was modified. Instead of optimizing the similarity measure within a registration box, we established a sequential priority-based optimization scheme. This scheme assumes that the alignment of a selected VOI might be prioritized against another VOI. Thus, the MI value of the first VOI in the priority queue is optimized with the resulting translation being set as input for the next iteration. The MI values of the resulting translation for all other VOIs are saved alongside. Then the MI function for the next VOI in the priority queue is optimized. The increase of the current VOI similarity and the decrease of the previously optimized VOIs are calculated. The new optimized translation is only accepted if an increase of the local similarity measure within the current VOI and only a minor decrease of a maximum of 1% of each already processed VOI are achieved. If these criteria are not fulfilled the old translation is kept.

## Results

### Characteristics of IGRT correction vectors

The mean values of expert-approved and calculated correction vectors for all patients and fractions are summarized in Table 1. Alongside, values for the corresponding Pearson’s correlation coefficient in comparison to the expert-approved corrections and the mean values of the absolute differences of all correction vectors per fraction Fi, Δ_BONY-RT_^Fi^, are presented. It should be noted, that even the smallest Pearson’s correlation coefficient in Table 1 at the sample number N = 757 is significant at the level of 0.01 (*p*-value = 0.00001). The critical value for the Pearson’s correlation coefficient at the same condition is <0.1. Expert-approved correction vectors, T_RT_, are distributed with one standard deviation of 3 mm in each direction. Correction vectors resulting from the bony match, T_BONY_, show a comparable distribution range, but only a low in-plane correlation with the expert-approved correction shifts. Correction vectors resulting from a registration with a small clipping box around the tPTV result in higher Pearson’s correlation coefficients in right-left (rl) and anterior-posterior (ap) direction compared to the bony match. However, the cranio-caudal component is reduced. The large registration box, T_pPTV_, leads to the strongest in-plane agreement with the expert-derived correction vectors and with the second highest z-correspondence. Table 1 also displays the results of a newly constructed registration approach, T_KNOWLEDGE_.

**Table 1:** IGRT correction vectors accepted by experts and proposed by competing automated registration methods

Correction type T_τ_	Correction vectors Mean ± SD [mm]			Pearson’s correlation coefficient PCC(T_τ,_ T_RT_)			Absolute differences Δ_τ-RT_ = |T_τ_^Fi^-T_RT_^Fi^| Mean ± SD [mm]		
	x	y	z	x	y	z	x	y	z
T_RT_	0.1 ± 3.0	0.2 ± 2.9	−0.3 ± 2.6	1	1	1	0	0	0
T_BONY_	0.3 ± 2.2	−0.5 ± 1.2	−0.1 ± 2.6	0.59	0.38	0.75	1.7 ± 1.7	2.1 ± 1.9	1.4 ± 1.2
T_tPTV_	0.3 ± 2.2	0.5 ± 2.6	0.1 ± 2.7	0.62	0.63	0.49	1.4 ± 1.8	1.5 ± 1.9	2.0 ± 1.8
T_pPTV_	0.3 ± 2.7	0.6 ± 2.2	−0.2 ± 2.5	0.89	0.79	0.70	0.9 ± 1.0	1.2 ± 1.4	1.5 ± 1.3
T_KNOWLEDGE_	0.1 ± 2.5	−0.3 ± 2.5	−0.1 ± 2.6	0.80	0.73	0.75	1.2 ± 1.2	1.4 ± 1.6	1.4 ± 1.2

*Fi* fraction i, *x* right-left direction, *y* anterior-posterior direction, *z* cranio-caudal direction, *SD* standard deviation

### Evaluation of trade-offs in alignment quality

**Fig. 1:**
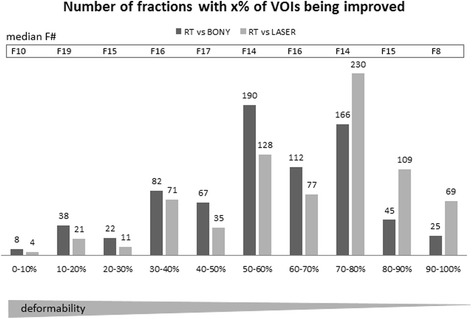
Histogram displaying the frequency of fractions with bad to good alignment of delineated structures: Number of fractions with volumes of interest (VOIs) that were improved in terms of an increased similarity value by the radiation therapists. Light (dark) grey colored bars indicate the improvement achieved by expert correction in respect to the laser-based (bony-based) positioning = expert-IGRT vs. non-IGRT (= expert-IGRT vs. bone-IGRT). The x-axis of the histogram describes the fraction of VOIs per patient (e.g. 50–60% interval holds the number of fractions, where the expert correction achieved an improvement in 50–60% of all delineated VOIs of the corresponding patient). The numbers above the bars resemble the height of the bar for better comparison. The fraction number Fi framed in the upper part of the graph indicates the median fraction number within the series in the corresponding dark grey bar (e.g. the dark grey bar with 50–60% of VOIs per patient experiencing a similarity improvement has the median fraction number F14)

Figure 1 shows the number of CBCTs separated according to the percentage of VOIs improving due to the correction of the expert. The interval dependency of the data reflects the deformability versus rigidity of the anatomy: It strongly indicates that most image content has changed rigidly if the alignment of 90-100% of delineated VOIs could be improved by the manual correction. In contrast, the improvement of the alignment of only several VOIs (e.g. 10–20% per patient) reflects stronger deformations in these fraction scans. The bars, colored light grey, show the fractions with expert-improvement T_RT_ in respect to the original laser-positioned anatomy T_LASER_. In T_LASER_ rigid offsets are not corrected, so most VOIs in most fractions benefit from the expert corrections: The distribution of light grey bars is shifted towards the right hand side of the histogram. The dark grey colored histogram shows fractions experiencing an improvement in respect to bony-corrected anatomy. This histogram reflects the trade-off in the alignment in presence of remaining deformations. The median fraction number of all contributing fractions into each bar, displayed in the framed box above the corresponding bars, indicates no visible time trend in deformation extent along the treatment course. An expected time trend indicating more deformations in later fractions would lead to a higher median fraction number in the first 0–30% intervals compared to the last 70–100% intervals, which is not observed.

**Fig. 2:**
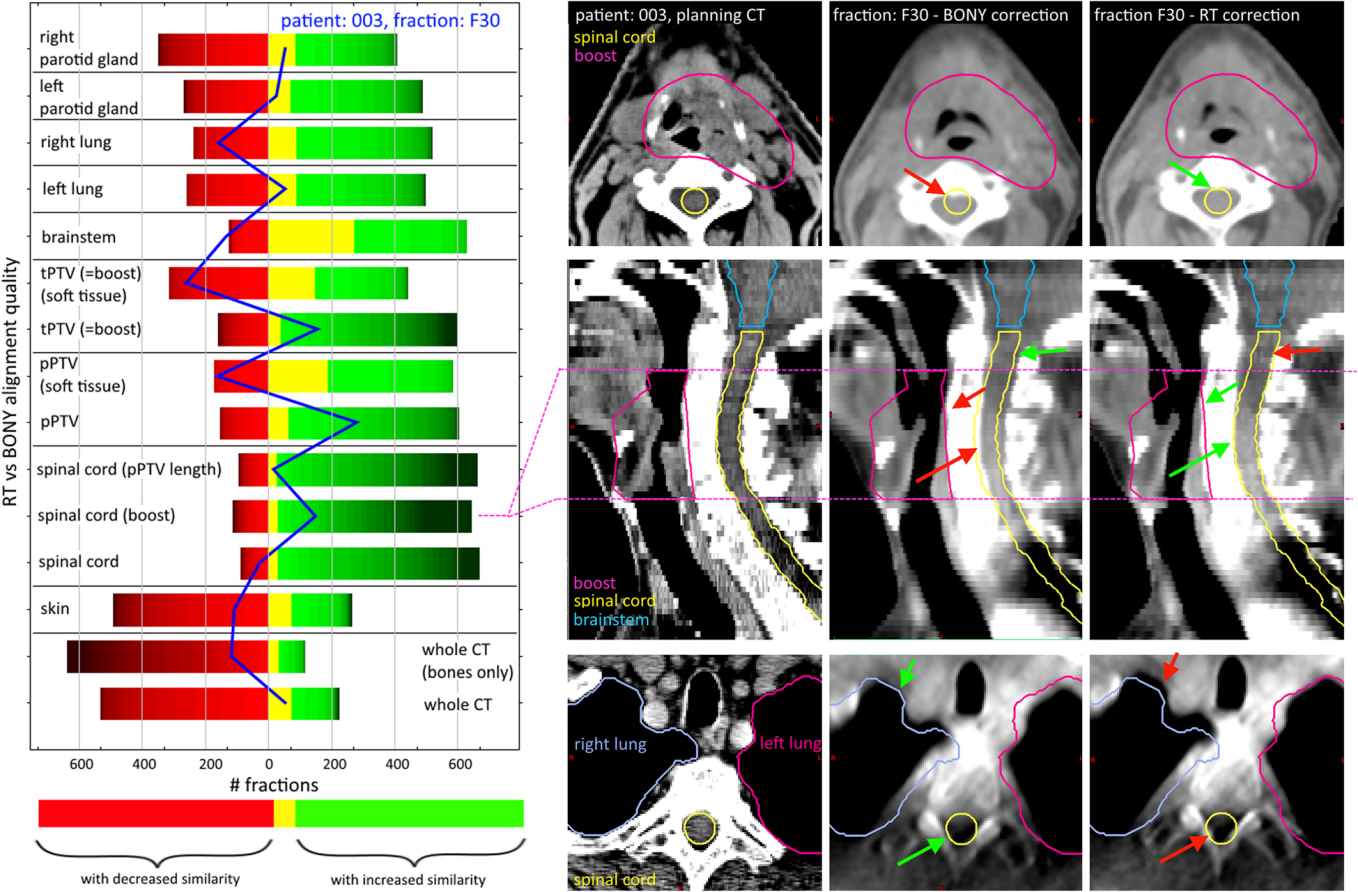
*Left panel:* Histogram displaying VOIs that increase (*green*) or decrease (*red*) their local similarity by the manual expert-correction in respect to the bony pre-match. The *blue line*, belonging to one exemplary fraction and patient, reflects the trade-off in the VOI alignment process. While for this selected patient geometry the structure alignment for spinal cord (boost) was improved, the whole spinal cord structure experienced a degradation of alignment. *Right panel*: CT views of the selected patient 003 visualizing the trade-off in the alignment quality of local structures. The VOIs indicate corresponding positions in the planning situation. The *dotted line* indicates the cc extension of the spinal cord (boost) substructure. The *green arrows* mark location with visible improvement of the alignment quality of the fraction scan in regard to the planning CT. The *red arrows* are the counterpart of the green ones, marking a decline of alignment quality

Improvement or decline of alignment quality separated according to all present VOIs is displayed in Fig. 2. Here, the improvement of the expert corrections T_RT_ in respect to T_BONY_ is presented to focus on the remaining deformations. The number of fractions with improved or declined VOI-similarity between the CBCT and the planning-CT is coded in green and red bars, respectively. The color intensity codes the extent of the improvement or degradation of the corresponding similarity measures, indicating only moderate changes in most of the fractions for all VOIs. The single zigzag-appearing line, representing this fraction (Fig. 2), reflects the trade-off for one exemplary fraction between the alignments of several structures. Most pronounced improvement, indicated by darker colors, is found in the alignment of the spinal cord and both PTVs. The scans belonging to the patient and fraction indicated in the blue line are displayed alongside in Fig. 2. Green arrows vs. red arrows mark the improvement vs. decline of the local alignment quality. Of course it has to be kept in mind that the whole 3D volume contributes to the overall localized similarity value not easy to demonstrate on selected 2D views.

A clustering analysis for all fractions with similar lines, like the exemplary zigzagged fraction in Fig. 2, was performed, grouping fractions with similar patterns. In the analyzed cohort a notable cluster formation based on expert versus bony corrections appeared, if the combination of only four VOIs was considered: pPTV, tPTV, brainstem, and spinal cord limited to the tPTV extension along the body axis, in the following labelled “spinal cord (boost)”. The prominent clusters are summarized in Table 2. Further clustering revealed the VOI priority present in our data set: spinal cord (boost), tPTV, pPTV followed by brainstem.

**Table 2:** Priorities for VOI alignment in expert-approved IGRT corrections resulting from clustering on combinations of VOIs with an improvement compared with the bony pre-match

Cluster label	Improvement – pattern {*S*_*VOIj*_}^*Fi*^				Number of fractions within cluster in %
	pPTV	tPTV	Brainstem	Spinal cord (boost)	
Cluster 2	1	1	1	1	39.4
Cluster 1	1	1	0	1	19.4
Cluster 6	1	1	1	0	5.9
Cluster 8	0	0	1	1	5.9
Cluster 14	0	1	1	1	5.4
Cluster 10	1	0	1	1	5.0
Cluster 7	0	0	0	1	3.9
Cluster 3	0	1	0	1	2.2
VOI priority	3	2	4	1	

*Fi* fraction i, *VOIj* volume of interest j

### Parametrization for the knowledge-based rigid registration scheme

Based on the priorities detected in the clustering process the sequence for priority-based optimization was determined as follows: First, the MI value in bones was optimized in order to achieve a bony pre-match. Then, the cranio-caudal-component of the resulting correction was locked and a subsequent rigid registration optimizing the MI, scored in i-th VOI of the priority queue, was performed. The priority queue was selected according to the clustering result: spinal cord (boost), tPTV, pPTV, brainstem, parotid glands, and upper lungs.

## Discussion

It is assumed that some criteria like dominant visibility (e.g. of bones), localization of dose gradients (e.g. around high-dose regions), knowledge about organ deformability or shrinkage (e.g. of parotid glands [12]) contribute to the IGRT correction of an experienced radiation therapist. In contrast, the rigid image registration does not ’know’ which of the structures should be aligned best. To identify measures for these criteria, similarity measures focused to VOIs in IGRT-corrected datasets were analyzed, based on the expert corrections. A new registration procedure was deduced to mimic the expert’s choice.

In this analysis, the radiation therapists considered it most important to align the high-dose region if a trade-off was necessary. This was reflected by high prioritization of the spinal cord (boost) structure and the tPTV. Corrections along the body axis can mainly be described by the bony pre-match (Table 1), indicating that also experts use bony structures as a prominent guiding aid. However, the high-dose region alone did not dictate the correction in all fractions, since the registration bounded to that structure (T_tPTV_, Table 1) showed the worst correlation among the analyzed corrections. In fact, in fractions with lesser deformations still the pPTV-bound registration performed comparable to the radiation therapists, showing highest correlation coefficient between the in-plane (rl, ap) correction components. Instead, the novel knowledge-derived registration method achieved a high z-correlation with the expert corrections, forced by the bony alignment, and still was capable to achieve high correlation coefficients in-plane.

The knowledge-based registration approach as presented here is still not fully optimized as it could be. As a first step, it is intended to demonstrate the construction of such an approach, but further knowledge-derived parameters could be included. It might be useful to stratify the expert-based IGRT corrections into groups with differing OAR-to-PTV-distances, to change the prioritization based on individual distances of dose gradients to OARs. In our data, a significant correlation between fractions with highest prioritization of the brainstem and a short distance to both high-dose regions was not detectable. This was due to the limited statistical power, if only fractions with a higher prioritization of the brainstem (=9%, 68 out of 757 CBCTs) are considered. Since this is a retrospective analysis and each correction was performed by one expert only, inter-observer variability could not be assessed. Also the limited number of analyzed patients did not allow for stratification according to individual experts.

Another limitation of this study due to the small number of patient data sets, is the lack of the division into a training cohort and a validation cohort for the knowledge-based registration approach. The validity of the derived specific knowledge-based registration method, of course, should be validated using a separate data set, which requires an additional similar cohort of the same size. Yet, our work is not intended to focus on the specific registration method but uses it for illustration on how to derive criteria in this question. In different institutions, experts might be trained to different priorities.

The outcome of this study is twofold: First, it presents a proof-of-principle for an approach to reveal human knowledge and criteria for human decisions in a multi-variate optimization problem, if desired. Such an approach can be used to extract information from an acquired big data set to enable automated cohort analysis. Second, it illustrates the derivation of a specific method to estimate an IGRT correction which enables a supervised learning approach. The advantage of the presented approach in comparison to previously proposed ones is that additional re-contouring [6] is not required. Once this approach is integrated onsite, manual expert corrections can be continuously analyzed by the proposed pipeline to re-adapt the knowledge-based registration scheme presented here. Embedding such an approach into rigid registration onsite is promising to provide a pre-match similar to the experts final match, so the necessity for manual correction is minimized. This can help to further reduce the time of the decision step [3], and it can also help to standardize the IGRT process. This standardization is essential, since variability in automatic registrations [8] and human interactive corrections limit the comparison of dosimetric measures in differently treated cohorts. While IGRT is associated with a PTV-margin reduction to 3–5 mm [1], the variability in IGRT corrections of 2–5 mm can have a considerable impact.

## Conclusions

Deriving optimal IGRT corrections in the presence of deformations is still challenging due to different trade-offs in the alignment of different structures. To replace expert-based interactive corrections, user knowledge needs to be incorporated into automated similarity optimization. In this work we propose how data in IGRT can be used to extract this knowledge and integrate it in the assessment of IGRT corrections, thus being capable to reproduce human decisions.
